# Fungal Biodeterioration Risk in Monastic Libraries without Climate Control

**DOI:** 10.3390/microorganisms12071450

**Published:** 2024-07-17

**Authors:** Katharina Derksen, Peter Brimblecombe, Guadalupe Piñar, Monika Waldherr, Alexandra Graf, Martin Haltrich, Pascal Querner, Katja Sterflinger

**Affiliations:** 1 Academy of Fine Arts Vienna, Institute of Natural Sciences and Technology in the Arts (INTK), Augasse 2-6, 1090 Vienna, Austria; 2 School of Environmental Sciences, University of East Anglia, Norwich NR4 7TJ, UK; p.brimblecombe@uea.ac.uk; 3 Department of Marine Environment and Engineering, National Sun Yat-sen University, Kaohsiung 80424, Taiwan; 4 Department of Applied Life Sciences/Bioengineering/Bioinformatics, FH Campus Wien, Favoritenstrasse 226, 1100 Vienna, Austria; 5 Abbey Library of Klosterneuburg, Stiftsplatz 1, 3400 Klosterneuburg, Austria; 6 Natural History Museum Vienna, 1. Zoology, Burgring 7, 1010 Vienna, Austria; 7 Institute of Zoology, University of Natural Resources and Life Sciences, Gregor-Mendel-Straße 33, 1180 Vienna, Austria

**Keywords:** monitoring, mold, indoor microclimate, metagenomics, historic building, books, Austria, cultural heritage, filamentous fungi, historic collections

## Abstract

Fungi have always posed an unquestionable threat to heritage collections worldwide. Now, in a future of climate change, biological risk factors may have to be considered even more than before. Models and simulations to assess possible impacts a changing outdoor climate will have on indoor environments and, in turn, on biodeterioration are still underdeveloped and require a more substantial data basis. This study aimed at filling some of these knowledge gaps through a broad-based approach combining microclimatic and microbiological monitoring in four historic libraries in Austria with an uncontrolled indoor climate: Altenburg Abbey, Melk Abbey, Klosterneuburg Monastery and the Capuchin Monastery in Vienna. Data were generated from thermohygrometric sensors, cultivation-dependent air- and surface sampling and further surface dust sampling for cultivation-independent analyses. Results gave insights on the status quo of microbiological loads in the libraries and outdoor–indoor relationships. Influences of the geographic location and room-use on corresponding indoor fungal profiles were identified. Lower fungal diversities were found at the most rural site with the strongest climatic fluctuations and extreme values than in the most urban, sheltered library with a very stable climate. Further, the humidity-stabilizing potential of large collections of hygroscopic materials, such as books, was also examined. Implications for a sustainable approach to prevent future biodeterioration are discussed, supporting the long-term preservation of these valuable historic collections.

## 1. Introduction

Fungi pose an unquestionable threat to cultural heritage collections. They can irrevocably damage or destroy historic objects, but also afflict building materials and present health risks [1]. Historic libraries are especially vulnerable to attack [2,3] as there are frequently constraints on controlling the indoor climate. Filamentous fungi particularly can cause great structural and aesthetic damage to these collections, mostly made up of readily degradable organic materials: leather/parchment, paper, starch, wood or photographic materials, all of which are, in addition, hygroscopic and already “pre-degraded” through age [3,4,5,6]. Stefan Michalski [7] once stated his thoughts on the matter bluntly (speaking specifically of humidity risks): “the single greatest risk worldwide from incorrect climate—mold”. It is a problem that is long recognized [8], where cold and damp conditions encouraged mold so were seen as a particular threat to books. Ongoing climate change is expected to exacerbate fungal attack by providing warmer conditions in humid environments [9,10,11,12,13] and accelerated biochemical reactions, especially in combination with a predicted increase in extreme weather events such as heavy rain and floods [14].

Previous studies have focused on the unique climate conditions inside historic buildings and, more specifically, those parameters concerning the preservation of cultural heritage collections often housed within them [15,16,17]. The ongoing discussion about suitable temperature and relative humidity values for their long-term conservation [7,18,19,20,21] becomes especially difficult in the case of historic buildings without any technological means of climate control. Many historic libraries fall into this category, which raises many questions about the current conservation state of the valuable, centuries-old objects, but also about their future. Attempts at actively controlling the indoor climate with HVAC systems, to comply with international guidelines and visitor comfort, potentially cause more harm to the historic objects, which had already acclimatized to natural fluctuations over centuries [22,23,24,25]. In monastic libraries without HVAC systems, the indoor microclimate is primarily influenced by the geographic location and exposure of the building, construction materials used, room air volume, windows, visitors and other factors. For the collections themselves (historic books and manuscripts, paintings, wooden furnishings, etc.), many more detailed factors are important that determine the microclimate around them—the microcosm relevant for biodeterioration. The degradation processes acting directly on the aged materials also depend on material composition, the (micro-) airflow around them, dust and other factors.

Research on the development of and proposals for suitable standard procedures for risk identification and damage prevention in historic libraries is already taking place [23,24,25,26,27], but very rarely accounts for both on-site microclimatic and biological data collection [28,29]. Furthermore, many studies carried out on fungi in the heritage environment have focused on single cases of infestations, specific objects or groups [30,31,32,33], but more systematic studies and a broader data basis are needed for better comparability and evaluation of future risks. Since the indoor environment is still a comparatively dry environment in temperate climates, xerophilic and xerotolerant species can be regarded as the most relevant threat to the objects here [34,35,36]. For example, in libraries and archives in particular, *Aspergillus halophilicus* (syn. *Eurotium halophilicum*) has been identified as an important agent of fungal infestation [37,38].

This study is part of a larger research project aimed at obtaining comprehensive data relating microclimatic parameters to fungal abundance and diversity in indoor spaces housing valuable natural and cultural heritage collections and archives. Such investigations are needed to improve comparability and risk assessment in these environments. The first comparative study conducted within this scope is presented here. Four monastic libraries were chosen, whose collections are subjected to entirely uncontrolled climatic conditions to analyze present fungal abundance and biodiversity in correlation to the indoor microclimate. For this purpose, a complementary approach was used including both the monitoring of microclimatic conditions during one year and the analysis of fungal communities through a combination of culture-dependent and -independent approaches (metagenomics). The data obtained are intended to provide a robust assessment of the current (and potential future) risk of fungal biodeterioration in these historic collections in view of predicted changes due to climate change.

## 2. Materials and Methods

The combination of methods applied for the generation and analysis of climatic and biological data is based on the methodology of a previous study [39]. It was adapted slightly and expanded for the analysis of the different locations investigated here, as shown in the following.

### 2.1. Description of Sites

**Figure 1:**
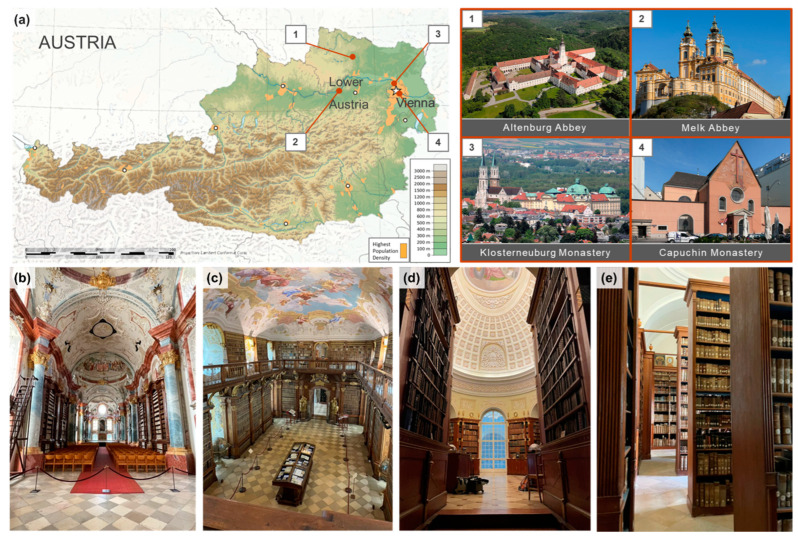
Overview of the four sites chosen for this study: (**a**) 1–4: their geographic location in Austria and outdoor views of the buildings, (**b**–**e**): interior of investigated rooms: Altenburg Abbey Library (ALT), Melk Abbey Library (MEL), Klosterneuburg Monastery Library (Kuppelsaal, KLO) and the Capuchin Monastery Library (CAP) [Map of Austria and outdoor views adapted from Wikimedia Commons; indoor photos K. Derksen].

For this study, four monastic libraries in the north-east of Austria were chosen for comparison, located in different environments from rural to urban (Figure 1). The libraries were constructed during the 18th and 19th centuries and today house valuable collections, from medieval Christian manuscripts to historic poem collections of the 19th century. More detailed descriptions of the libraries and interiors are given later in the Discussion section. Altenburg Abbey’s library (ALT) is in the most exposed and rural location of the four, built on a hilltop surrounded almost entirely by forests and meadows. Melk Abbey’s library (MEL) and Klosterneuburg Monastery’s library (KLO) can be categorized as intermediate—both buildings also stand exposed on hilltops, but in the middle of smaller cities surrounded by green landscapes, although Klosterneuburg lies directly on the outskirts of Vienna. The Capuchin Monastery´s library (CAP) is located in the most urban setting, situated in the city center of Vienna, with almost no green spaces in its vicinity. In the following, the four investigated library rooms are referred to by the abbreviations introduced above.

### 2.2. Indoor and Outdoor Climate Data

**Figure A1:**
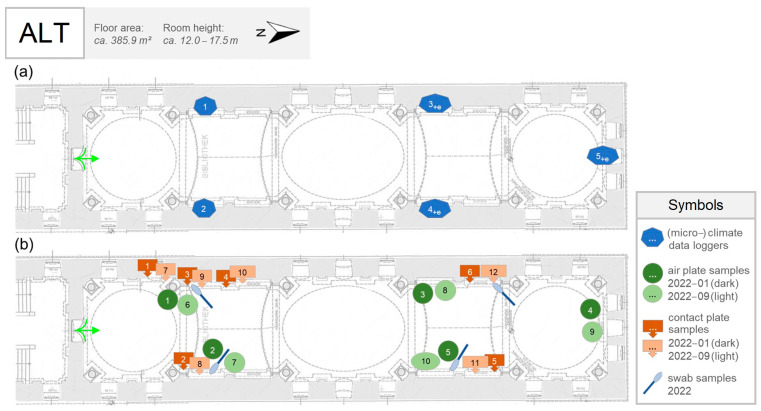
Overview of the library room at Altenburg Abbey (ALT): room measurements, orientation, locations of climate sensors (**a**) and biological sampling points (**b**). A more detailed description of symbols is given in the box in the lower right corner.

In total, 25 thermohygrometric sensors (Datalogger calibrated by the supplier, MostraLog, Long Life for Art, Eichstetten, Germany) were placed throughout the rooms, in microclimatic niches such as between or behind books, on top of shelves (Figure A1, Appendix A). Some also had additional external sensors reaching down to the floor. Monitoring of temperature (T) and relative humidity (RH) was undertaken for the duration of one year, from July 2021 to July 2022, at 15 min intervals.

Further, material moisture measurements were taken inside the rooms with a moisture meter (P 5201, PeakTech^®^, Ahrensburg, Germany) four times per year: November/December, January/February, April/May and July. Different material categories were measured, namely the walls, the wooden bookshelves and the books themselves, and at three different heights (10 cm above the floor, at 130 cm and at 190 cm).

Outdoor climate data for the four locations were compiled and downloaded from the GeoSphere Austria Website (Zentralanstalt für Meteorologie und Geodynamik, ZAMG). Data from the geographically nearest and most representative ZAMG weather stations were chosen for all four sites: Station Horn for Altenburg Abbey, Station Melk for Melk Abbey, Station Wien Hohe Warte for Klosterneuburg Monastery and Station Wien Innere Stadt for Capuchin Monastery (https://data.hub.geosphere.at/group/stationsdaten, accessed on 24 November 2023; time period: 1 July 2021 to 15 August 2022; variables: T, RH) and further processed in MS Excel^®^ 2022.

### 2.3. Microbiological Data

#### 2.3.1. Sampling

**Figure A2:**
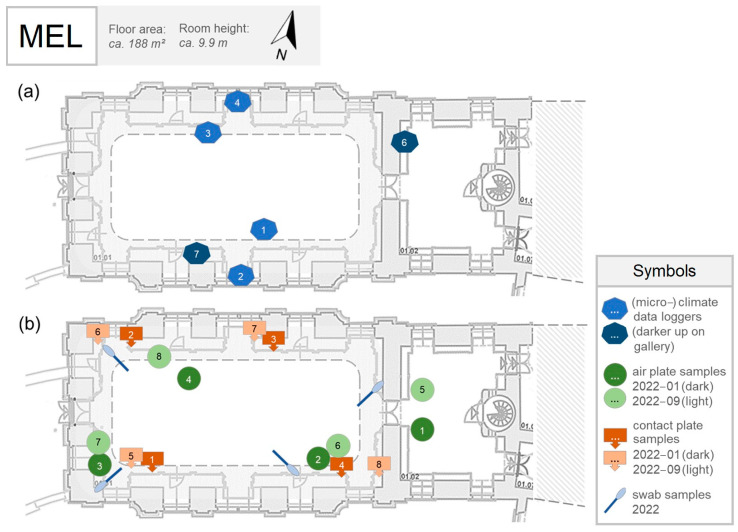
Overview of the library room at Melk Abbey (MEL): room measurements, orientation, locations of climate sensors (**a**) and biological sampling points (**b**). A more detailed description of symbols is given in the box in the lower right corner.

**Figure A3:**
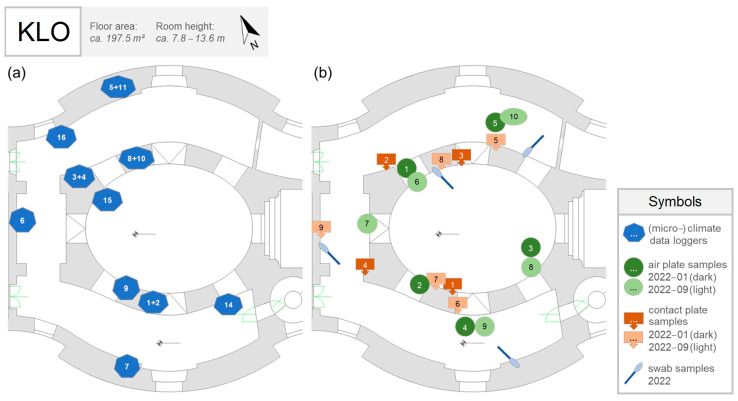
Overview of the library room at Klosterneuburg Monastery (KLO): room measurements, orientation, locations of climate sensors (**a**) and biological sampling points (**b**). A more detailed description of symbols is given in the box in the lower right corner.

**Figure A4:**
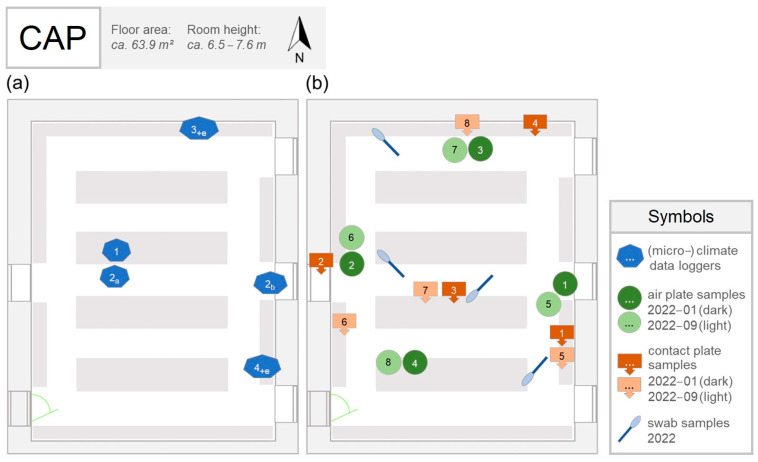
Overview of the library room at Capuchin Monastery (CAP): room measurements, orientation, locations of climate sensors (**a**) and biological sampling points (**b**). A more detailed description of symbols is given in the box in the lower right corner.

Microbiological sampling campaigns took place in January and September 2022, always during the morning, when it was assumed that air was least disturbed. Weather conditions during the winter and summer sampling days are provided in Supplementary Table S1. Fungal sampling points were chosen, where possible, in accordance with the positions of climate sensors (Figure A1, Figure A2, Figure A3 and Figure A4, Appendix A). A combination of three complementary sampling methods was applied for the analysis and identification of the fungi present: (i) active air sampling, (ii) surface sampling with contact plates and (iii) further surface sampling of dust using dry sterile cotton swabs for the molecular analyses.

Malt Extract Agar (MEA) and Dichloran-Glycerol-Chloramphenicol Agar (DG18) were used for cultivation, both for air (ø 90 mm) and contact plates (ø 55 mm). In total, between 8 and 10 air samples plus two outdoor references (device MAS-100 Eco^®^, MBV, Stäfa, Switzerland; sampling volume 100 L, sampling height 1.5 m above the floor) and 8–12 contact plate samples were taken per location and season, depending on room size and complexity. Contact plates were not applied to books but directly to surfaces of the room furnishings: windowpanes, paneling, bookshelves in front of or next to books (further procedures see Section 2.3.2). Four swab samples per library room were taken from settled dust over an area of 50 cm^2^, depending on available surfaces (from shelves, tops of bookshelves or interior decorations such as pillars or wooden paneling) and pooled after DNA extraction in the lab (see Section 2.3.3).

#### 2.3.2. Cultivation Plate Analysis

After incubation at room temperature for a minimum of 7 days, fungi present in the indoor air and on surfaces were directly analyzed quantitatively and qualitatively (absolute counts and morphological identification to genus level; identification literature: [40,41,42,43,44,45]) by microscopy (Olympus SZ40 and Leica DM500). Only for a small number of frequently recurring species that were of interest due to potential health risks or threats to the historic materials were isolations and identification down to species level attempted. All counts are given in colony-forming units (CFUs) and expressed as concentrations for air samples (CFUs/m^3^) and contact samples (CFUs/m^2^).

#### 2.3.3. Metagenomic Analysis

Total DNA was extracted from each swab sample using the FastDNA™ Spin Kit for Soil and corresponding protocol (MP Biomedicals, Irvine, CA, USA). For each historic library, the four DNA samples were pooled after extraction and concentrated for 1.5 h in a vacuum concentrator (Savant SpeedVac DNA-130 Vacuum Concentrator, Thermo Fisher Scientific, Waltham, MA, USA), followed by measurements with a Qbit 2.0 fluorometer (Qbit™ dsDNA HS Assay-Kit, Thermo Fisher Scientific, Waltham, MA, USA).

A long-amplicon sequencing approach was chosen to obtain a broad overview of the diversity of the fungal community present, focusing on the fungal Internal Transcribed Spacer (ITS), corresponding to the ITS1 and the ITS2 regions and the 5.8S rRNA gene between them (PCR primers used: ITS1 (forward) 5′-TCCGTAGGTGAACCTGCGG, and ITS4 (reverse), 5′-TCCTCCGCTTATTGATATGC) [46,47]. A first round of PCR using the Promega PCR Master Mix 2X in a C1000 Touch™ Thermal Cycler (BIO-RAD, Feldkirchen, Germany) to amplify the target sequences was performed as follows: The premixed PCR Mastermix, 2X (50 units/mL of Taq DNA polymerase supplied in a proprietary reaction buffer (pH 8.5), 400 µM dATP, 400 µM dGTP, 400 µM dCTP, 400 µM dTTP, 3 mM MgCl_2_) was diluted to 1X and 0.5 pmol/µL of each primer (stock: 50 pmol/µL) were added. A total of 4 µL of template DNA was added and the reaction mix adjusted to a total volume of 50 µL with nuclease-free H_2_O. The following program was used: 3 min at 95 °C, followed by 35 cycles of 1 min at 95 °C, 30 s at 55 °C and 1 min at 72 °C, with a final extension step of 5 min at 72 °C.

The Nanopore sequencing platform (barcoding- and library preparation kits, sequencing devices and software were all from Oxford Nanopore Technologies, Oxford UK) was selected for this metagenomic study. The PCR Barcoding Kit (SQK-PBK004) was used as described by Tichy et al. [48] but adapted for fungi: customized tailed primers ITS1(F) and ITS4(R) (New England Biolabs, Ipswich, MA, USA) from a stock solution of 2.5 pmol/µL were used for the Barcoding PCR reaction with a corresponding annealing temperature of 55 °C and an extension temperature of 65 °C for 45 s. Barcodes 01–04 were assigned to the four historic libraries, followed by pooling of all four samples for the preparation of a single sequencing library. After priming and loading of the DNA library onto a SpotON Flow cell (Mk I R9, FLO-MIN106), which was previously quality checked using the MinKNOW™ software 21.11.7., sequencing was performed in a MinION Mk1C device, with a run duration of 48 h and concurrent high accuracy basecalling (Guppy 5.1.13). All read data have been submitted to the NCBI public database (BioProject accession number PRJNA904284).

For sequence analysis, first, remaining adapters were removed from basecalled reads, and chimeric sequences were split using porechop (version 0.2.4). Then, reads were filtered with NanoFilt (version 2.8.0) to remove low-quality read ends (40 bases trimmed at 5′ and 3′ ends) and to obtain quality scores (QS) > 9 as well as read lengths between 400 and 900 bases (expected amplicon length). After filtering, the median QS were between 11.9 and 13.6, representing error rates between 6.46 and 4.37%.

Metataxonomic classifications were performed with Emu (version 3.4.4) using the provided pre-built UNITE general fasta v8.3 fungi database, containing the RepS/RefS of all Species Hypotheses (SH; OTUs defined in a way that they correspond to fungal species level, as used by UNITE) (created 10 May 2021). Visualization of the classification results was performed in R (4.3.1) using the packages pheatmap, tidyverse and RColorBrewer. Relative abundance cut-offs were set to 0.1% or 0.5% on the shown taxonomic level, and all classifications below that threshold set to “unidentified”.

### 2.4. Statistics

A range of statistical methods were used, although these needed to recognize that the sample sizes were sometimes small and were ordinal in nature with many zeroes. We used box and whisker plots to present CFU data. The box is bounded by the 25th and 75th percentiles, with the median denoted by the central line in the box. The whiskers represent the range of all other points, except those that are deemed as outliers. Because of the ordinal nature of much of our data, non-parametric tests were adopted. The Kendall rank correlation (statistic τ) was used to establish the extent of ordinal association and used the online software (https://wessa.net/rwasp_kendall.wasp, accessed on 21 January 2024). The significance of the difference between the distributions was tested with software available at Vassarstat (http://www.vassarstats.net/, accessed on 7 February 2024). Non-independent samples involving matched sets of data used either the Wilcoxon signed-rank or Friedman tests depending on the number of sets compared. The difference among the distributions of independent samples utilized the Kruskal–Wallis test (a non-parametric equivalent of ANOVA) with the post hoc analysis using Dunn *p*-values, further adjusted by the Benjamini–Hochberg FDR method from Astatsa (https://astatsa.com/, accessed on 15 January 2024).

## 3. Results

### 3.1. Outdoor Climate and Indoor Microclimate

#### 3.1.1. Temperature and Relative Humidity

**Figure 2:**
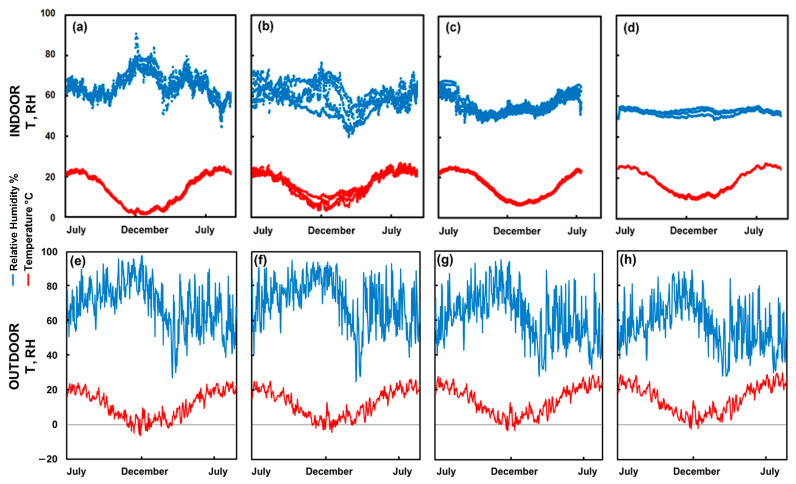
Daily average temperature T (°C, red line) and relative humidity RH (%, blue line) from July 2021 to July 2022 indoors and outdoors: (**a**,**e**) ALT and outdoor (Station Horn), (**b**,**f**) MEL and outdoor (Station Melk), (**c**,**g**) KLO and outdoor (Station Vienna Hohe Warte) and (**d**,**h**) CAP and outdoor (Station Vienna City Center). Records all start beginning of July 2021 until approximately August 2022.

Outdoor climate values from the nearest weather stations, as described in the previous section, are presented in Table 1, in comparison to the indoor microclimate within the libraries as annual averages. An ANOVA test for the temperature and relative humidity (both outdoors and indoors) suggests that these four meteorological parameters are not all drawn from the same populations (*p* < 0.001), indicating that the measured outdoor and indoor climates differ significantly between the four locations. Respective indoor and outdoor climate variabilities throughout the seasons are shown in Figure 2.

The average indoor temperatures vary slightly among the libraries. The variation in temperature for individual libraries is largely due to the changing seasons. They are quite cold in the winter with ALT almost down to 0 °C (Figure 2a). As the summer temperatures in all the rooms are typically 21–22 °C, it is the winter that affects the annual average.

The average relative humidity differs among the libraries. It was high in the winter, especially at ALT, which seems notably cold and damp in comparison to the other libraries, with winter temperatures just a few degrees above zero and RH close to 80%. In contrast to this, KLO was drier in the winter with RH as low as 50% at times. It shows very little variation at CAP.

#### 3.1.2. Water Content

**Figure 3:**
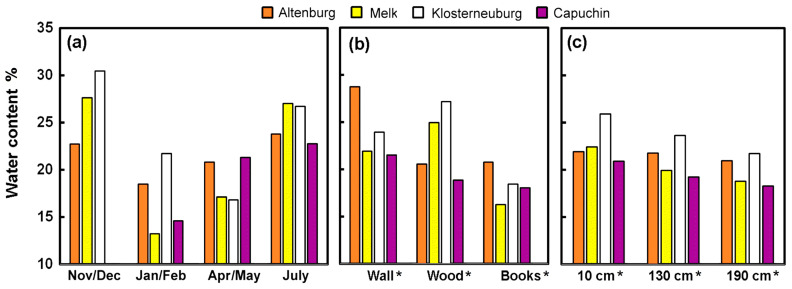
(**a**) Average water content (%) of surfaces in the libraries at various times of year, (**b**) average water content of materials and (**c**) average water content of all material surfaces at different heights above the floor. Note that values for November/December 2022 are missing at Capuchin. “*” denotes high significance (*p* < 0.0001) of differences between measured materials and measurement heights.

As the location of the measurements changed slightly throughout the year, samples were uncorrelated, so the means were compared using the average values in a Friedman test, which suggested the mean ranks were November/December 3.8, January/February 1.3 (the lowest), April/May 1.8 and July 3.3, with *p* < 0.017. The January/February and April/May values appear lower than those of November/December and July (Figure 3a).

The water content from the three surfaces could readily be paired, so the differences between measurement sets from all seasons and libraries (441 triplets of data) were assessed using a Friedman test. This showed the wall ranked with the highest water content (mean rank 2.5) compared with wood (1.9) and books (1.6), with significant differences (*p* < 0.0001), which aligns with the situation presented in Figure 3b. A similar analysis using data from all the libraries showed that the samples collected at 10 cm had the highest water content (mean rank 2.3), compared with the samples at 130 cm and 190 cm, which ranked 2 and 1.7, with differences significant at *p* < 0.0001. Although the changes are small, the decreasing water content with height is borne out by Figure 3c.

### 3.2. Microbiological Data

#### 3.2.1. Cultivation Data

Samples of the air and selected surfaces in the four libraries were collected and incubated as shown in the Methods section. The focus of the quantitative and qualitative analysis presented below was on the generally most abundant genera or taxonomic groups, which are also of greatest interest in historic interiors. The main identified organisms therefore belong to the genera *Aspergillus*, *Penicillium*, *Cladosporium* and *Alternaria*. The findings are presented in Table 2, which gives an overview of CFU concentrations collected from the air or detected on surfaces, with numbers from both MEA and DG18 combined. The lower parts of Table 2 show the concentrations of viable airborne fungal cells and those deposited on surfaces, sampled in winter and in summer at each location. For comparison, the values obtained in the winter and summer samplings were averaged.

Data from the indoor air samples were first analyzed in comparison to the outdoor air reference samples (see also Supplementary Table S2). The average totals of CFU counts in the air samples collected outside of the four libraries (ALT, MEL, KLO, CAP) in winter were 715, 205, 900 and 390 CFUs/m^3^ and in Summer 480, 1570, 2670 and 620 CFUs/m^3^. The average values for each of the main genera were: *Aspergillus* spp. (2.5/m^3^), *Penicillium* spp. (9.44/m^3^), *Cladosporium* spp. (54/m^3^), *Alternaria* spp. (5.5/m^3^), *Fusarium* spp. (1.7/m^3^), *Epicoccum* spp. (0.13/m^3^) and *Mucoromycota* (0.06/m^3^). Examining the data for all the samples collected, these four were ranked for the most abundant CFU counts: *Cladosporium* spp. (mean rank 3.9), *Penicillium* spp. (mean rank 2.7), *Alternaria* spp. (mean rank 2.1) and *Aspergillus* spp. (mean rank 1.3, the lowest), with a Friedman test indicating that the concentrations were significantly different (*p* < 0.0001).

**Figure 4:**
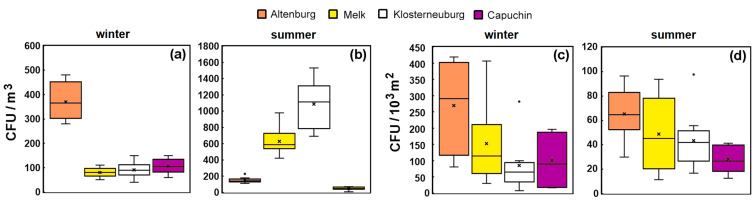
Total number of CFUs collected inside the four libraries from the air (CFUs/m^3^) in (**a**) winter and (**b**) summer, and surfaces (CFUs/10^3^ m^2^) in (**c**) winter and (**d**) summer. “×” denotes the mean. “·” indicates outliers.

The total CFU counts in the outdoor air appear generally greater in the summer, although not outside of ALT. However, removing this library, a Wilcoxon signed-rank test gave results suggesting that the summer samples were, overall, significantly greater (*p*_2_~0.05). In the outdoor air, the summer medians are 480, 1570, 2670 and 620 CFUs/m^3^ outside ALT, MEL, KLO and CAP, respectively, with notably high summer concentrations outside KLO and MEL. Median counts in winter were 715, 205, 900 and 390 CFUs/m^3^, outside of ALT, MEL, KLO and CAP, respectively—all being higher than the median values for the indoor air samples as shown in Figure 4a. A slight but positive correlation was found between indoor and outdoor concentrations in the reference samples of the four main genera (Kendall τ = 0.38, *p*_2_ < 0.005). The Wilcoxon signed-rank test suggests that indoor concentrations are typically lower than outdoor concentrations (*p*_2_ < 0.0005) with a notable exception of the high concentrations of *Aspergillus* sp. at KLO in the summer (see Section 4).

The CFU counts from indoor air samples (testing across all libraries for the four most abundant genera) were not significantly higher in summer than in winter. Closer examination showed that summer air concentrations were indeed higher at KLO and MEL (Wilcoxon signed-rank test *p*_2_~0.01), while at CAP and ALT, the reverse was true, although at CAP, this arose because of high *Penicillium* concentrations in winter air. The median concentrations of CFUs counted from the indoor air samples in winter at the libraries were: ALT 365, MEL 80, KLO 90 and CAP 105 CFUs/m^3^, as shown in Figure 4a. The most rural of the libraries (ALT) had the highest counts, but in the summer, it was KLO and MEL (Figure 4b), and the median values in summer at CAP and ALT were lower than in winter. *Cladosporium* spp. were, overall, the most abundant, then came *Penicillium* and *Aspergillus* spp., followed by the occasional detection of *Alternaria* spp., *Fusarium* spp., *Epicoccum* spp. and *Mucoromycota*. Among the least abundant taxa detected, and therefore included in the “Others” column of Table 2, are, for example, other *Ascomycota* such as *Paecilomyces*, *Trichoderma*, *Chaetomium, Exophiala* and *Aureobasidium*, as well as the Basidiomycete *Wallemia.*

Somewhat similar patterns were found in the data obtained from the surfaces investigated through contact plate observations (Figure 4c,d). A slight (*p*_2_~0.1) but positive correlation (Kendall *τ*~0.21) was found between the four most common genera collected in air and on surfaces at the same time in the various libraries. In all but one of the campaigns, *Cladosporium* spp. dominated the samples collected on contact plates, indicating its dominance on the library surfaces.

#### 3.2.2. Metagenomic Data

For a more exhaustive characterization of the mycobiome associated with dust deposited on the surfaces, targeted ITS long-amplicon sequencing was carried out with Nanopore technology and the generated data further processed as described in Section 2.3.3.

The yield of DNA extracted from the cotton swabs used to collect the dust was, as expected, very low: ALT 0.092 ng/µL, MEL 0.272 ng/µL, KLO 0.402 ng/µL and CAP 0.024 ng/µL. However, after amplification of the ITS regions, a sufficient concentration was obtained to prepare DNA libraries in which all four samples were barcoded. They were then loaded and sequenced in a single flow cell. Total reads generated in the sequencing were 3,997,603, comprising a total yield of 1.04 Gb. For the single barcodes/sites, reads ranged from 446,226 to 1,347,870 (see Supplementary Table S3). Median lengths of the reads finally used for assignment after quality filtering were between 456 and 547 bp, aligning with the targeted amplicons’ length. The highest amount of data was recovered from ALT, with 172,860 assigned reads, followed by samples MEL, CAP and the fewest assigned reads from KLO.

**Figure 5:**
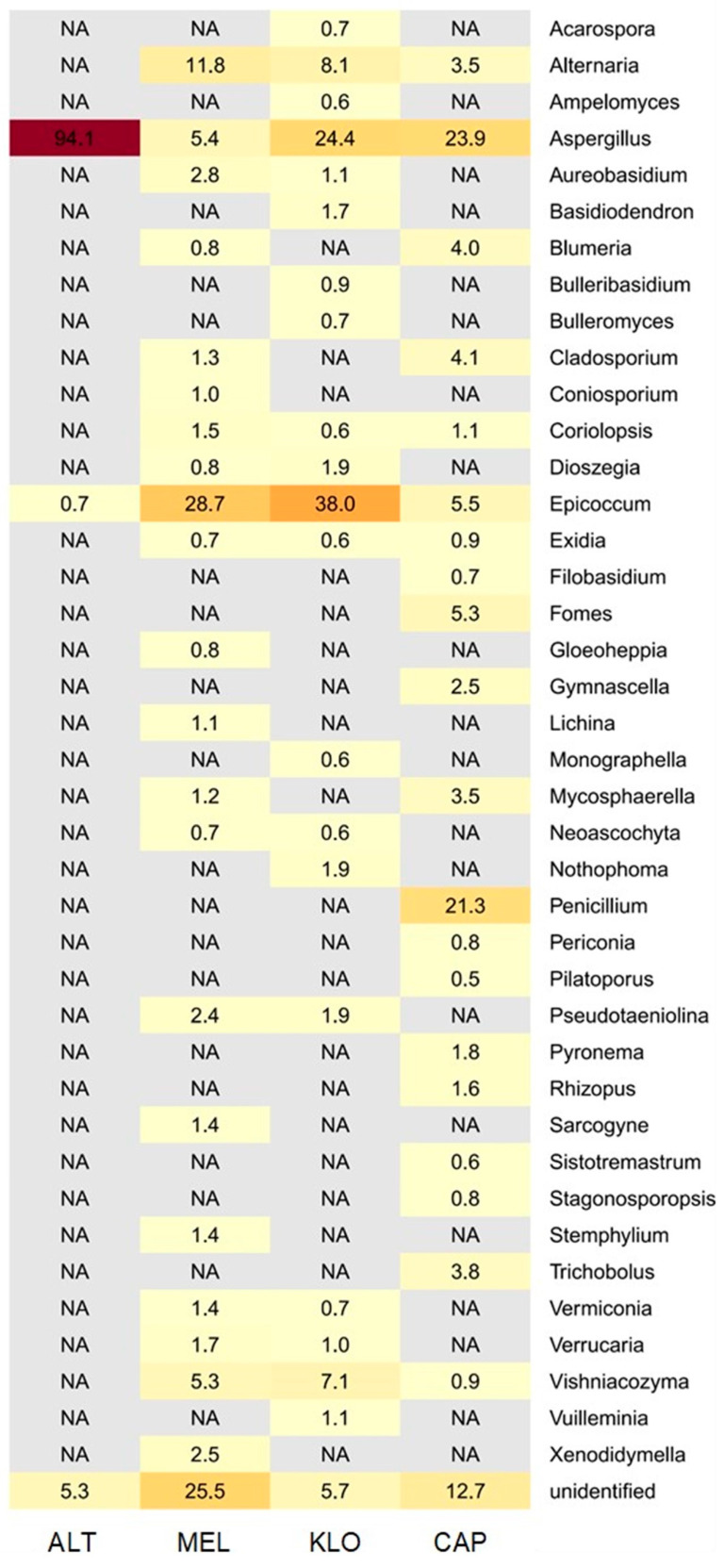
Heatmap displaying the relative abundance (%) of fungal communities (OTUs at genus level, abundance cut-off 0.5%) within the four library rooms, as identified by metagenomic analysis. Colors correspond to the abundance values, the darker the color, the higher the relative abundance. “Unidentified” denotes classifications identified only to a higher taxonomic level. “NA” denotes classifications below the 0.5% abundance threshold or under the detection limit within that sample.

**Figure 6:**
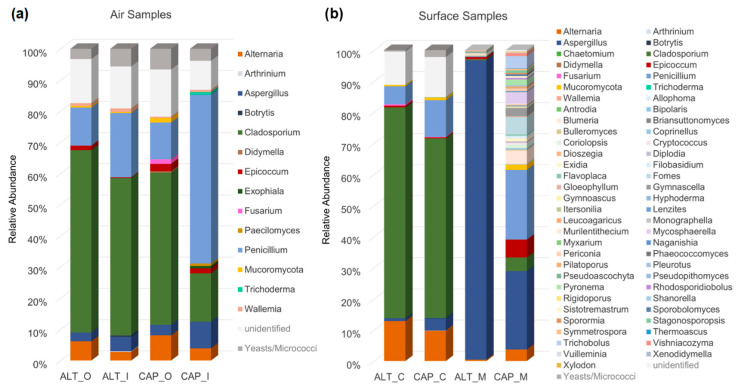
Comparison of fungal profiles from all samples collected at ALT and CAP. (**a**) Bar chart displaying relative abundance (%) of identified and unidentified fungal taxa (max. genus level resolution) from outdoor (ALT_O/CAP_O) and indoor (ALT_I/CAP_I) air samples (MEA and DG18, winter and summer combined for each location); (**b**) bar chart displaying relative abundance (%) of fungal communities determined on indoor surface samples: ALT_C/CAP_C from contact plates (genus level; MEA and DG18, winter and summer combined for each location), ALT_M/CAP_M display the metagenomic data (OTUs; max. genus level resolution; abundance cut-off 0.001%).

Phylogenetic assignments from DNA sequencing data revealed high diversities at an abundance level of less than 0.5%, but for the purpose of a simplified presentation, Figure 5 gives an overview of identified OTUs at the genus level with an abundance cut-off of 0.5%. At the phylum level, *Ascomycota* clearly dominate over all the sampled locations, with the main representatives of the overall most abundant genera being (in descending order) *Aspergillus*, *Epicoccum, Penicillium* and *Alternaria*, followed by *Basidiomycota* (overall most abundant genus identified as *Vishniacozyma*). A few reads were further assigned to *Mucoromycota* (e.g., *Rhizopus*). Between the four sites, ALT was clearly dominated by *Aspergillus* spp. (94.1%), MEL and KLO by *Epicoccum* spp. (28.7% and 38.0%), and to a lesser extent, *Penicillium* spp. (5.4% and 24.4%), but at CAP, *Aspergillus* (23.9%) and *Penicillium* (21.3%) were the most abundant taxa. Less abundant genera (>0.001%) are also listed in Figure 6. The main differences in the diversity profiles of the four libraries can broadly be described as follows: ALT is distinctly different from all others; MEL and KLO show higher similarities to each other and to CAP, but CAP again has a more singular fingerprint.

#### 3.2.3. Comparison of Fungal Profiles

The results of culture and metagenomic analyses across all libraries revealed certain similarities. Figure 6 gives a further, more detailed insight into the identified fungal diversity profiles and summarizes them with the example of a comparison between the library rooms ALT and CAP, which showed the most distinctive profiles of all four sites (see also Supplementary Figure S2).

At ALT, indoor air samples reflected the outdoor air strongly, with *Cladosporium* spp. clearly dominating and, overall, the same identified factions of taxa, while, in contrast, the results at CAP showed a very different picture indoors (Figure 6a). *Cladosporium* was found in lower numbers here, while *Penicillium* dominated; *Aspergillus* also made up a higher proportion of counts and further genera such as *Trichoderma* or *Paecilomyces* were identified only in the indoor air samples. The samples from MEL and KLO gave intermediate results, similarly to the description of the metagenomic results.

Surface samples presented another pattern (Figure 6b). Those from contact plates resembled the air samples to a certain extent, regarding the fractions of main genera, but did not show the same clear differences between the four sites. In the metagenomic profiles, a slight overlap was found with the results from cultivation-based analyses but, mainly, other additional taxa were identified here. It is also again highlighted that, in ALT, the diversity at the genetic level appears to be much lower than in CAP.

## 4. Discussion

At the outset of this study, four historic libraries were selected to represent one of four distinct building categories as part of a larger project assessing the impact of climate change on museum pests. Despite their numerous similarities such as uncontrolled indoor climate, comparable building envelopes, age and collection materials, it was hypothesized that differences in microclimate and fungal diversity or abundance profiles would emerge due to their varying geographic locations and surroundings (rural vs. urban), among other potential factors. This hypothesis is supported by the insights gained through the combined methodological approach of microclimatic and microbiological monitoring applied in this study, which are discussed below, together with additional findings.

### 4.1. Historical and Spatial Context

Initially, it is essential to delve into the history of the libraries and their interiors to better contextualize our findings. The two oldest buildings among the four were completed by the Benedictines in Altenburg in 1742 and in Melk in 1735. Subsequently, the Augustinian Canons established their new location in Klosterneuburg in 1837, followed by the Capuchins in Vienna in 1841.

In Altenburg Abbey, a freestanding north–south-oriented library wing was erected, based on Joseph Munggenast’s plans. Due to the descending terrain, it rests on a substantial substructure (crypt) and rises above the surrounding forests [49]. The hall, divided into five sections, boasts three flat domes adorned with frescoes by Paul Troger and Jakob Zeiller [50]. Presently, the library houses around 7000 volumes of its 25,000-book collection in ornate wooden bookshelves along the outer walls [51].

Similarly, the Baroque tract of the Melk library, planned by Jakob Prandtauer, is prominently situated in the northern part of the monastery atop a rock plateau facing the Danube [52]. The main hall, along with an adjoining smaller room, are decorated with frescoes by Paul Troger and Gaetano Fanti on their vaulted ceilings. Both rooms are two stories high and connected by a continuous gallery. The inlaid wooden furniture, holding a collection of around 16,000 historic volumes, is placed against the walls and, in the main room, conceals two of the five windows, accessible only through hidden doors [53].

The main hall of the Klosterneuburg Abbey Library is situated within a dome structure, built in the 1730s, initially intended as an entrance to a monumental staircase [54]. In 1837, Josef Kornhäusel established this library hall, the Kuppelsaal, in the space beneath the cupula [39]. The baroque architecture features a central rotunda surrounded by arcades on three sides. Open bookshelves resting on enclosed lower cabinets together accommodate approx. 40,000 volumes [55].

Around the same time as the library construction in Klosterneuburg, Vienna’s deteriorating 17th-century Capuchin Monastery in the city center was replaced by a new building designed by imperial–royal architect Joseph Baumgartner [56]. In 1841, the late-classicist library was inaugurated. Apart from three windows in the northern wall, all walls are entirely furnished up to the ceiling with fluted lisenas and wooden shelves, connected by central transverse shelves [57]. They hold ca. 13,000 books.

All four investigated library rooms are enveloped by a similar building structure: massive stone and brick walls, stone-tiled floors, and all have windows, only differing in number, size, orientation and exposure.

### 4.2. Humidity Buffering Capacity of Historic Books

The finding that the measured relative humidity (RH) did not correlate with the material moisture content was not surprising, as material moisture seldom directly correlates with room RH. It strongly depends on the surface materials involved (e.g., natural wood, oiled, lacquered or painted surfaces, bare or salt-affected walls, books with parchment or paper bindings, etc.). This underscores the necessity of conducting RH monitoring for conservation purposes as close to the objects as possible. Notably, ALT exhibits generally higher RH levels, while KLO shows the highest overall moisture content in its materials (walls, wooden bookshelves and historic books). Statistical analysis, however, showed no clear correlation of moisture content with interior RH.

As the thermohygrometric analyses shed light on a clear discrepancy between the annual fluctuations of T and those of RH within the four libraries (see also Figure 2), this was investigated further. A similar observation was also made by Andretta et al. [58] and Kupczak et al. [59] who found that sizeable paper collections can have a measurable impact on humidity stability within a closed space. The assumption, that the large collections of very hygroscopic materials (mainly the historic books) within these indoor spaces might play a role, was tested by comparing the room air volumes with the numbers of books stored within. Book numbers were presumed a suitable parameter as they also infer the available area of wooden shelving and amount of buffering material. Numbers were determined by correspondence with librarians at the sites, literature research and own calculations. While straightforward for ALT, MEL and CAP, the calculation for KLO took into account that a significant portion of the collection in this room (the Kuppelsaal) is stored in double rows on open shelves or within closed cupboards. For better comparability with the other three libraries, the number of books standing only in front rows on open shelves was approximated. Final numbers used for calculations were: ALT 7000, MEL 16,000, KLO 20,000 and CAP 13,000 books. For the calculation of air volumes, only the minimum ceiling heights were considered (all rooms have cupula structures, see also Figure A1, Figure A2, Figure A3 and Figure A4, Appendix A). A Buffer Index (B.I.) is proposed as a possible explanation for the different levels of RH buffering observed within the four libraries. The B.I. equals the number of books per m^3^ of the respective indoor air volume of rooms (m^3^). Resulting indices calculated for each library were: 1.5 (ALT), 8.6 (MEL), 13.0 (KLO) and 31.3 (CAP), thus attributing the highest humidity buffering capacity to CAP. In direct comparison, these findings correspond well with the different observed annual indoor RH fluctuations (see Figure 2a–d; Supplementary Figure S1 gives a visual summary of the described correlations). Further investigation also showed that the amount of water in the books in the libraries by far exceeded that in the air, suggesting their great potential buffering capacity.

### 4.3. Microbiological Findings

Overall, the complementary results obtained from the fungal analyses underscore the advantages of combining cultivation-dependent and -independent approaches for studying microbial communities [60,61,62].

The two distinct examples shown in Figure 6 were selected because they exhibited the clearest differences in fungal community compositions between indoor and outdoor air samples (see also Supplementary Figure S2). At Altenburg Abbey, as the site most exposed to its surrounding environment, indoor air samples reflected the outdoor air much more strongly than in the other three locations. This suggests a more direct exchange of air through the larger, more airy windows exposed to strong winds on the hilltop where Altenburg Abbey stands. This contrasts, for example, with the much fewer, more compact and well-shielded windows at the Capuchin Monastery, with its library room facing only a small courtyard as outdoor space. The other two sites take on intermediate places. Inside Melk Abbey’s library wing, the profiles of indoor and outdoor air were quite similar to those in Altenburg, which may be explained by the regular exchange of air through automatically opening doors from the constant flow of visitors. Again, in contrast, the more shielded windows and low visitor frequency inside the Kuppelsaal at Klosterneuburg Monastery can explain the greater differences in air sample composition there. It is noteworthy that the high numbers of *Aspergillus* sp. found in summer air samples at KLO (potentially indicating an outbreak, as discussed in [39]) were attributed to a singular event.

Although the same clear distinctions between the sites were not observed in the contact plates, they offer an important insight into the pool of viable fungi present on surfaces and therefore into the potential risk of growth, should favorable conditions arise. As presented in Section 3.2.2, the metagenomic analyses, however, revealed distinctive differences in the fungal profiles of the four libraries. In direct comparison, again following the pattern already described earlier, the different fungal DNA “fingerprints” at the sites may be attributed to their general location and surroundings. ALT and CAP seem to have the most distinct diversity profiles; MEL and KLO adopt intermediate positions here. The shift from low diversity at ALT to higher values towards CAP follows the expected pattern of lower diversities where a more fluctuating climate with more extreme values exerts a higher selective pressure on the (microbial) communities and of higher diversities in more constant environments. A higher diversity at MEL may further be explained by its large numbers of daily visitors and regular exchange with the outdoor air through automatically opening doors, while the other three sites are much more secluded in that sense.

One striking difference between cultivation and molecular samples was the absence of *Fusarium* in the metagenomic data, while all other main genera that were found in cultivation plates were also identified by metagenomic assignments. Additionally, *Aspergillus halophilicus* (syn. *E. halophilicum*), a common culprit in paper-based collections, was not found in the cultivation plates as expected due to its strong xerophilic nature, and its presence could not be unequivocally confirmed by the metagenomic approach used.

### 4.4. Microclimate and Fungal Biodeterioration Risk

The investigated indoor spaces, housing valuable historic collections, lack active climate control and have a supply of historic organic substrates, dust and old building materials as substrates. Moreover, their not completely airtight windows allow for the exchange of air with the outdoor environment. These conditions likely contribute to the abundant CFU counts observed in this study, some of which exceeded acceptable limits specified in international guidelines for fungal loads in everyday indoor spaces [63,64]). However, to date, there is no known problem of microbial biodeterioration in these libraries. Although simplified, the calculations described in Section 4.2 imply a clear connection between books/m^3^ of air volume in the rooms and buffering of indoor RH fluctuations throughout the year. It could be the explanation why, even though RH values reached up to 65–80% at times (by museum standards, a nonideal climate for preservation), no instance of mold growth was observed in any of the libraries.

This is a fact which is striking, as we indeed found high numbers of—viable but seemingly dormant—CFUs. In addition, several fungal taxa were identified whose species are well-known potential biodeteriogens in libraries, archives and museums (including, but not limited to, *Alternaria* spp., *Aspergillus*(/*Eurotium*) spp., *Cladosporium* spp., *Penicillium* spp. and *Trichoderma* spp.). While RH levels were often conducive to fungal growth in theory, the constantly fluctuating microclimate on the surfaces of the historic organic materials in these uncontrolled indoor climates may hinder sustained fungal growth. Therefore, it is proposed that the timeframe of humid conditions necessary for spore germination and growth, a crucial factor in mold development [65], is never quite reached on the materials. This suggests that, even with ongoing climate change, high-tech, energy-intensive HVAC systems are not the only option to explore for keeping biological deterioration processes at bay. In addition, while this important variable could not be investigated within the scope of this study, air flow rates are suspected to play a crucial role here as well and should be included in future research.

## 5. Conclusions

This research aimed to give an insight into potential current and future risks of biodeterioration, and to support decision makers in finding sustainable and efficient preventive solutions for collections. To this end, the required data were generated from parallel indoor climate monitoring and fungal sampling from air and surfaces.

Evidently, even though indoor RH varies greatly in some locations, including short-term peaks in unsuitably or dangerously high ranges (i.e., suitable for microbial growth according to collection climate guidelines), the mean material moisture of the objects and interiors remains relatively low over longer time periods—low enough to prevent present spores from germinating and causing an infestation. The buffering capacity of the interior and collection materials therefore seems to act in a way that the water involved never becomes biologically available for long enough. It could prove worthwhile to follow up on these observations by examining this connection of moisture availability and the time factor more closely, specifically concerning xerotolerant and xerophilic fungal species relevant for collections.

These results have a further, broader socio-economic impact, suggesting that, at least from a microbiological point of view (which to a large extent dictates given upper RH limits of climate guidelines in preventive conservation), maybe the focus can shift back towards more simple solutions rather than complicated technical fixes for minimizing biodegradation risk. Creating good air flow, regular (dry) cleaning of collection spaces, maintenance of buildings, monitoring and emergency planning and preparedness, as countless cases of mold infestations occur due to water damage or long response times, are paramount to continue to keep fungal growth risk to a minimum.

Nevertheless, physico-chemical degradation processes always act on objects of art and cultural heritage in parallel with biological factors and follow slightly different rules. They are, of course, also highly important to consider for preventive conservation strategies. This complex discussion over an optimal climate for collections has spanned many decades, and is still ongoing. The present research aims to add a further, microbiological view to this multi-facetted problem while advocating for more sustainable, cost- and energy-efficient approaches and solutions to protect our heritage in a future of climate change.

## Figures and Tables

**Table 1:** Outdoor and indoor climate data for all sites: Annual averages of temperature (T) and relative humidity (RH) outside of the buildings and inside the investigated library rooms.

Location		Altenburg Abbey	Melk Abbey	Klosterneuburg Monastery	Capuchin Monastery
Average T (°C)	Outdoor	11.1 ± 7.9	12.2 ± 7.8	13.6 ± 8.0	14.7 ± 8.2
	Library	12.2 ± 7.9	15.3 ± 6.2	15.9 ± 6.5	18.0 ± 5.8
Average RH (%)	Outdoor	69.1 ± 13.7	71.0 ± 14.0	62.4 ± 14.4	57.2 ± 13.6
	Library	66.1 ± 6.1	59.2 ± 6.4	55.4 ± 4.5	52.4 ± 1.4

**Table 2:** Averaged total counts of colony forming units (CFUs) collected at each of the four libraries from all indoor air and surface samples in winter (W) and summer (S), both MEA and DG18; average CFUs for specific fungal taxonomic groups of interest determined from all indoor air and surface samples at respective sites and seasons. Note: most abundant genera are listed explicitly, “Others” includes further, less frequently found *Ascomycota* (e.g., *Chaetomium*, *Paecilomyces*, *Trichoderma*), *Basidiomycota* (e.g., *Wallemia*), as well as unidentifiable colonies, sterile colonies and micrococci.

	Total CFUs	*Aspergillus*	*Penicillium*	*Cladosporium*	*Alternaria*	*Fusarium*	*Epicoccum*	*Mucoromycota*	Others
Indoor air [CFUs/m^3^]									
ALT-W	370.0	19.0	84.0	213.0	10.0	0	0	1.0	24.0
ALT-S	152.0	3.0	14.0	27.0	2.0	0	0	0	69.0
MEL-W	81.3	11.3	27.5	16.3	1.3	0	0	2.5	23.8
MEL-S	630.0	15.0	42.5	253.8	45.0	2.5	15.0	3.8	222.5 **
KLO-W	91.0	15.0	21.0	40.0	3.0	0	0	0	7.0
KLO-S	373.0 *	28.0 *	53.0	172.0	4.0	1.0	1.0	5.0	46.0
CAP-W	106.3	7.5	76.3	17.5	0	0	0	0	8.8
CAP-S	48.8	6.3	11.3	7.5	6.3	0	2.5	0	13.8
Indoor surfaces [CFUs/m^2^]									
ALT-W	27 × 10^4^	1813	15,063	138,125	34,438	1063	375	875	>19,688
ALT-S	7 × 10^4^	750	1563	50,938	1188	63	1125	313	>6688
MEL-W	15 × 10^4^	1016	3594	61,641	23,047	391	1016	313	>60,938
MEL-S	5 × 10^4^	781	2656	26,094	2031	0	547	313	>15,781
KLO-W	8 × 10^4^	3984	8906	17,422	2109	625	78	1797	>3906
KLO-S	5 × 10^4^	1797	78	20,547	0	78	0	234	>20,938
CAP-W	10 × 10^4^	1875	10,234	52,109	7891	469	0	469	>9375
CAP-S	3 × 10^4^	2266	2266	9219	2344	78	313	391	>8906

* Outlier (high *Aspergillus* sp. count in summer) removed; ** many micrococci.

## Data Availability

Publicly available data are given as URLs; metagenomic data are available at the NCBI public database (https://www.ncbi.nlm.nih.gov/, NCBI BioProject accession number PRJNA904284); and other data collected during the project are available on application to KD.

## Supplementary Materials

The following supporting information can be downloaded at: https://www.mdpi.com/article/10.3390/microorganisms12071450/s1, Table S1: Outdoor weather conditions during winter and summer sampling days. Table S2: Averaged total counts of colony-forming units (CFUs) from all outdoor air samples collected at each of the four libraries in winter (W) and summer (S), both MEA and DG18; average CFUs for specific fungal taxonomic groups of interest determined from all outdoor air samples at respective sites and seasons. Note: The most abundant genera are listed explicitly; “Others” includes further, less frequently found *Ascomycota* (e.g., *Chaetomium*, *Paecilomyces*, *Trichoderma*) and *Basidiomycota* (e.g., *Wallemia*), as well as unidentifiable colonies, sterile colonies and micrococci. Table S3: Results of ITS Sequencing and Quality Control (raw reads), Figure S1: Visualization of proposed “Buffer Index” (B.I), as a direct comparison between annual indoor RH fluctuations (a–d) and air volume of rooms vs. books calculated per m^3^ of air volume (e–h). Figure S2: Comparison of fungal profiles from all samples collected at ALT, MEL, KLO, CAP. (a) Bar chart displaying relative abundance (%) of identified and unidentified fungal taxa (genus level resolution) from outdoor (“_O”) and indoor (“_I”) air samples (MEA and DG18, winter and summer combined for each location); (b) bar chart displaying relative abundance (%) of fungal communities determined on indoor surface samples: Samples “_C” from contact plates (genus level resolution, MEA and DG18, winter and summer combined for each location); (c) “_M” displays the metagenomic data (OTUs, max. genus level resolution, abundance cut-off 0.001%).
