# Supplementary material for: Fungal Biodeterioration Risk in Monastic Libraries without Climate Control
Source: Microorganisms. 2024 Jul 17;12(7):1450. doi: 10.3390/microorganisms12071450 (PMC11279154; doi:10.3390/microorganisms12071450)
Supplement: Supplementary file 1 [file microorganisms-12-01450-s001.zip › microorganisms-3044928-supplementary.pdf]

# Fungal Biodeterioration Risk in Monastic Libraries without Climate Control

**Katharina Derksen [1,*], Peter Brimblecombe [2,3], Guadalupe Piñar [1], Monika Waldherr [4], Alexandra Graf [4], Martin Haltrich [5], Pascal Querner [6,7] and Katja Sterflinger [1,*]**

## Supplementary Materials

**Table S1**: Outdoor weather conditions during winter and summer sampling days.

**Table S2**: Averaged total counts of colony forming units (CFU) from all outdoor air samples collected at each of the four libraries in winter (W) and summer (S), both MEA and DG18; average CFU for specific fungal taxonomic groups of interest determined from all outdoor air samples at respective sites and seasons. Note: Most abundant genera are listed explicitly, "Others" includes further, less frequently found *Ascomycota* (e.g. *Chaetomium, Paecilomyces, Trichoderma*), *Basidiomycota (e.g. Wallemia)*, as well as unidentifiable colonies, sterile colonies and micrococci.

**Table S3:** Results of ITS Sequencing and Quality Control (raw reads).

**Figure S1:** Visualization of proposed "Buffer Index" (B.I), as a direct comparison between annual indoor RH fluctuations (a-d) and air volume of rooms vs. books calculated per $m^3$ of air volume (e-h).

**Figure S2:** Comparison of fungal profiles from all samples collected at ALT, MEL, KLO, CAP. (a) Barchart displaying relative abundance (%) of identified and unidentified fungal taxa (genus level resolution) from outdoor ("_O") and indoor ("_I") air samples (MEA and DG18, winter and summer combined for each location); (b) Barchart displaying relative abundance (%) of fungal communities determined on indoor surface samples: Samples "_C" from contact plates (genus level resolution, MEA and DG18, winter and summer combined for each location); (c) "_M" display the metagenomic data (OTUs, max. genus level resolution, abundance cut-off 0.001 %).

**Table S1**

| Location | Conditions | T [°C] | RH [%] |
|---|---|---|---|
| Altenburg Abbey-W | light rain, windy (ca. 40km/h) | 1 | 70 |
| Altenburg Abbey-S | cloudy, light wind (ca. 14 km/h) | 13 | 70 |
| Melk Abbey-W | sunny, light wind (ca. 15km/h) | 3 | 80 |
| Melk Abbey-S | light rain, light breeze (ca. 5km/h) | 19 | 83 |
| Klosterneuburg M.-W | cloudy, windy (ca. 45km/h) | 3 | 65 |
| Klosterneuburg M.-S | cloudy, light wind (ca. 12km/h) | 24 | 50 |
| Capuchin Monastery-W | sunny, windy (ca. 40km/h) | 6 | 50 |
| Capuchin Monastery-S | sunny/cloudy, light wind (ca. 15km/h) | 19 | 70 |

**Table S2**

| | Total CFU | *Aspergillus* | *Penicillium* | *Cladosporium* | *Alternaria* | *Fusarium* | *Epicoccum* | *Mucoromycota* | Others |
|---|---|---|---|---|---|---|---|---|---|
| **Outdoor air [CFU/m³]** | | | | | | | | | |
| Altenburg Abbey-W | 715 | 30 | 115 | 375 | 20 | 0 | 5 | 0 | 120 |
| Altenburg Abbey-S | 480 | 10 | 15 | 245 | 30 | 0 | 10 | 5 | 110 |
| Melk Abbey-W | 205 | 15 | 40 | 30 | 20 | 5 | 0 | 10 | 95 |
| Melk Abbey-S | 1570 | 70 | 360 | 780 | 100 | 10 | 45 | 10 | 185 |
| Klosterneuburg M.-W | 900 | 5 | 85 | 480 | 25 | 0 | 0 | 0 | 75 |
| Klosterneuburg M.-S | 2670 | 25 | 15 | 1900 | 160 | 105 | 0 | 0 | 100 |
| Capuchin Monastery-W | 390 | 35 | 85 | 175 | 25 | 5 | 5 | 10 | 50 |
| Capuchin Monastery-S | 620 | 10 | 40 | 340 | 60 | 10 | 25 | 5 | 125 |

**Table S3**

| Barcode | Sample | Total Bases | Reads | Median Length | Median QS |
|---|---|---|---|---|---|
| **BC01** | KLO | 216,976,247 | 1,035,761 | 197 | 10.3 |
| **BC02** | CAP | 313,824,625 | 1,347,870 | 198 | 10.1 |
| **BC03** | MEL | 298,151,586 | 1,167,746 | 202 | 10.3 |
| **BC04** | ALT | 211,868,629 | 446,226 | 395 | 10.8 |

**Figure S1**

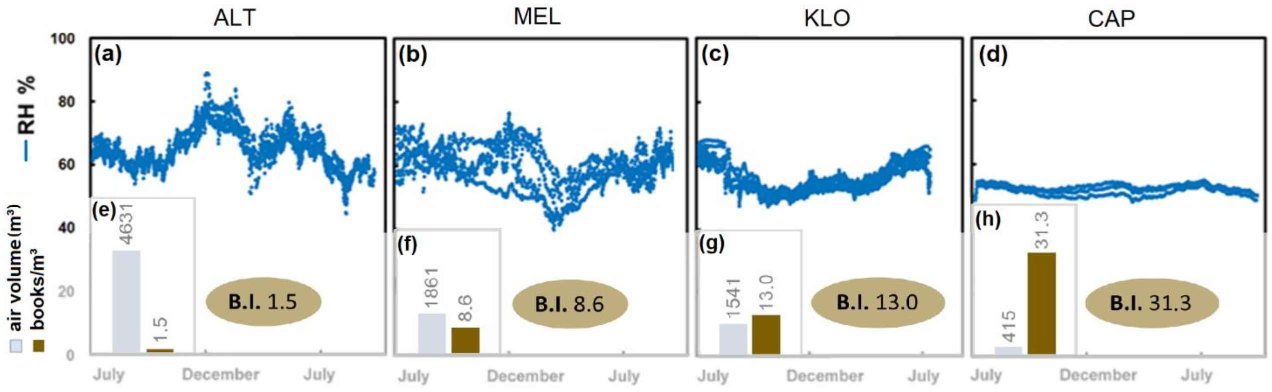

**Figure S2**

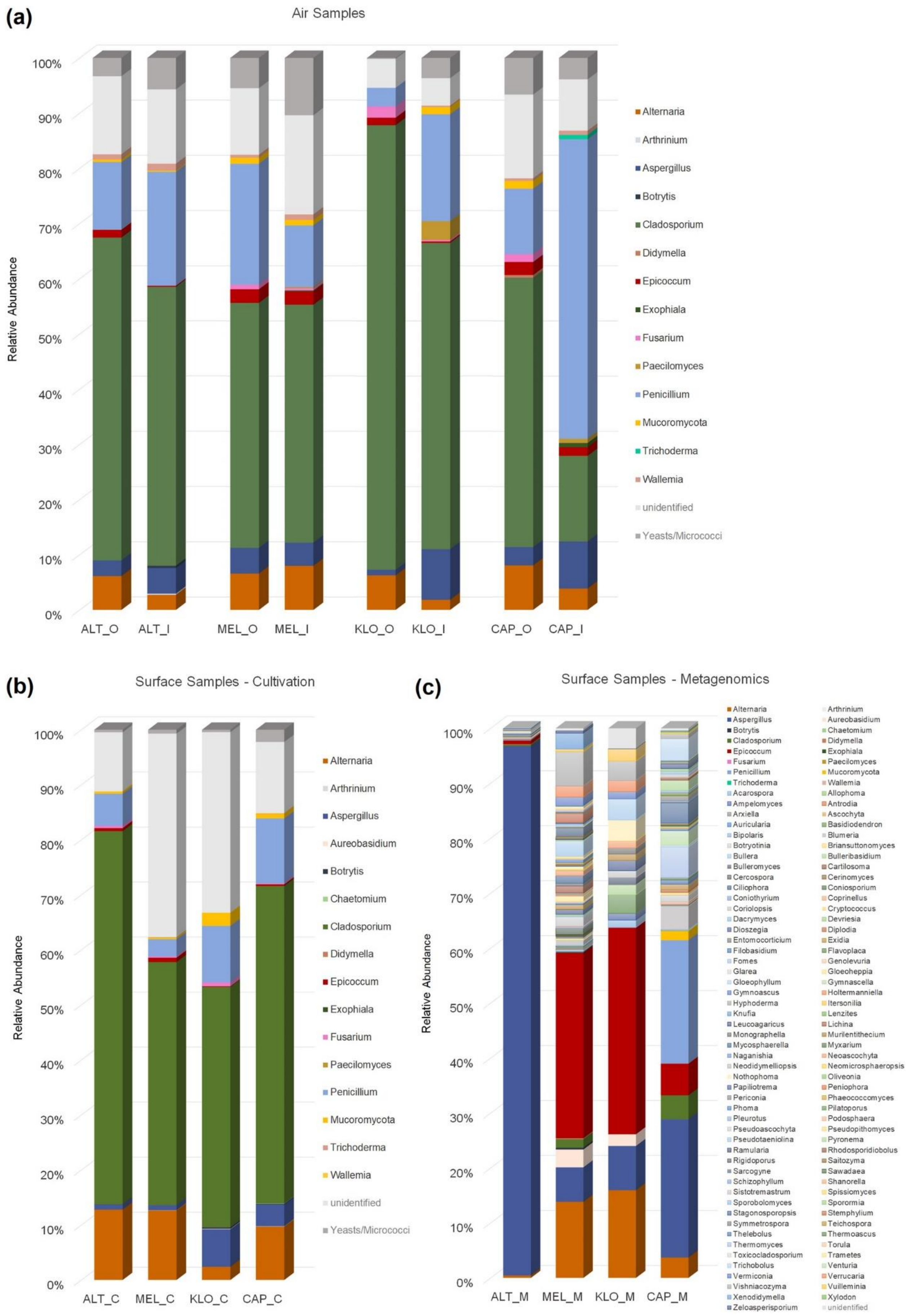
(a)
Air Samples
Relative Abundance
0%
10%
20%
30%
40%
50%
60%
70%
80%
90%
100%
ALT_O
ALT_I
MEL_O
MEL_I
KLO_O
KLO_I
CAP_O
CAP_I
Alternaria
Arthrinium
Aspergillus
Botrytis
Cladosporium
Didymella
Epicoccum
Exophiala
Fusarium
Paecilomyces
Penicillium
Mucoromycota
Trichoderma
Wallemia
unidentified
Yeasts/Micrococci
(b)
Surface Samples - Cultivation
Relative Abundance
ALT_C
MEL_C
KLO_C
CAP_C
Alternaria
Arthrinium
Aspergillus
Aureobasidium
Botrytis
Chaetomium
Cladosporium
Didymella
Epicoccum
Exophiala
Fusarium
Paecilomyces
Penicillium
Mucoromycota
Trichoderma
Wallemia
unidentified
Yeasts/Micrococci
(c)
Surface Samples - Metagenomics
Relative Abundance
ALT_M
MEL_M
KLO_M
CAP_M
Alternaria
Arthrinium
Aspergillus
Aureobasidium
Botrytis
Chaetomium
Cladosporium
Didymella
Epicoccum
Exophiala
Fusarium
Paecilomyces
Penicillium
Mucoromycota
Trichoderma
Wallemia
Acarospora
Allophoma
Ampelomyces
Antrodia
Arxiella
Ascochyta
Auricularia
Basidiodendron
Bipolaris
Blumeria
Botryotinia
Briansuttonomyces
Bullera
Bulleribasidium
Bulleromyces
Cartilosoma
Cercospora
Cerinomyces
Ciliophora
Coniosporium
Coniothyrium
Coprinellus
Coriolopsis
Cryptococcus
Dacrymyces
Devriesia
Dioszegia
Diplodia
Entomocorticium
Exidia
Filobasidium
Flavoplaca
Fomes
Genolevuria
Glarea
Gloeoheppia
Gloeophyllum
Gymnascella
Gymnoascus
Holtermanniella
Hyphoderma
Itersonilia
Knufia
Lenzites
Leucoagaricus
Lichina
Monographella
Murilentithecium
Mycosphaerella
Myxarium
Naganishia
Neoascochyta
Neodidymelliopsis
Neomicrosphaeropsis
Nothophoma
Oliveonia
Papiliotrema
Peniophora
Periconia
Phaeococcomyces
Phoma
Pilatoporus
Pleurotus
Podosphaera
Pseudoascochyta
Pseudopithomyces
Pseudotaeniolina
Pyronema
Ramularia
Rhodosporidiobolus
Rigidoporus
Saitozyma
Sarcogyne
Sawadaea
Schizophyllum
Shanorella
Sistotremastrum
Spissiomyces
Sporobolomyces
Sporormia
Stagonosporopsis
Stemphylium
Symmetrospora
Teichospora
Thelebolus
Thermoascus
Thermomyces
Torula
Toxicocladosporium
Trametes
Trichobolus
Venturia
Vermiconia
Verrucaria
Vishniacozyma
Vuilleminia
Xenodidymella
Xylodon
Zeloasperisporium
unidentified
